# Predictive Value of Adenoviral Load for Bronchial Mucus Plugs Formation in Children with Adenovirus Pneumonia

**DOI:** 10.1155/2022/9595184

**Published:** 2022-08-08

**Authors:** Li Peng, Silan Liu, Tian Xie, Yu Li, Zhuojie Yang, Yongqi Chen, Liangji Deng, Han Huang, Xiaofang Ding, Min Chen, Lin Lin, Sangzi Wei, Lili Zhong

**Affiliations:** Hunan Provincial Key Laboratory of Pediatric Respirology, Pediatric Medical Center, Hunan Provincial People's Hospital (The First Affiliated Hospital of Hunan Normal University), Changsha 410000, China

## Abstract

**Background:**

The study aimed to explore risk factors for bronchial mucus plugs (BMP) formation in children with adenovirus (AdV) pneumonia.

**Methods:**

A retrospective study was conducted on children with AdV pneumonia who underwent bronchoscopy from January 2019 to December 2019. Children were divided into the BMP group and the control group, depending on whether BMP was formed or not. The clinical information and treatment proposals of the two groups of children were counted and analyzed via multiple logistic regression analysis, ROC curve analysis, and correlation analysis.

**Results:**

Among 453 patients with AdV pneumonia, 185 (40.84%) were in the BMP group. Among all the cases, there were 188 patients with a single AdV infection, including 64 (34.04%) in the BMP group and 124 (65.96%) in the control group. The incidence of dyspnea, poor spirits, mixed infections, and other symptoms in the BMP group was higher than in the control group. Children in the BMP group had a longer heat range. C-reactive protein (CRP), lactate dehydrogenase (LDH), D-dimer (DD), and AdV load levels were higher in the MBP group. AdV load, *Mycoplasma* coinfection, DD, heat range, and LDH were independent risk factors for BMP, among which AdV load was the most significant (AUC = 0.819). AdV load was positively correlated with other risk factors, respectively. AdV load and heat range were independent risk factors for BMP patients with a single AdV infection.

**Conclusion:**

AdV load might have important clinical value in predicting BMP development in AdV pneumonia.

## 1. Introduction

Pneumonia is a common acute respiratory infection. The pathogens of pneumonia include bacteria, viruses, *Mycoplasma* pneumonia, etc. [1]. Adenovirus (AdV) infection accounts for 5% to 19% of childhood pneumonia [2–4]. The proportion of infection by different pathogens and the most recent citations are given in Supplementary Table 1. AdV pneumonia is characterized by many complications, sequelae, and a high case fatality rate. In the acute stage of AdV infection, inflammation can cause bronchial and bronchiolar mucosal edema, congestion, and necrosis and block the lumen to form bronchial mucus plugs (BMP) [5]. BMP formation is considered a serious complication of AdV pneumonia and an important reason for the formation of respiratory failure [6].

BMP is an endogenous bronchial foreign body formed by inflammation, hemorrhage, necrosis, and abnormal bronchial mucus secretion, followed by mucus accumulation and removal obstacles [7]. If it is not cleared in time, it will lead to partial or complete ventilatory dysfunction of the lungs, causing breathing difficulties, life-threatening situations, and even respiratory failure. In addition, various BMP and peeling of the mucosal epithelium can induce granulation tissue to remove these endogenous foreign bodies, which may be the pathological basis of bronchiolitis obliterans (BO) in the late stage of AdV pneumonia [8].

Zhang et al. [9] showed that prealbumin levels, glucocorticoid use time, C-reactive protein (CRP), and lactate dehydrogenase (LDH) levels are independent risk factors for BMP formation, while Xuefeng et al. [10] believe that age greater than 5 years and high IL-10 and IFN-*ɤ* have important predictive value for BMP formation. However, the above research subjects are children with *Mycoplasma pneumoniae* pneumonia, and there is no large sample report on the study of AdV pneumonia-BMP. AdV load is positively associated with the severity of pneumonia. AdV load can significantly increase the risk of severe pneumonia [11]. However, the specific mechanism is unclear. The study aims to analyze the predictive effects of AdV load in the BMP formation of children with AdV pneumonia to reduce the occurrence of sequelae of AdV pneumonia in early clinical diagnosis and treatment.

## 2. Materials and Methods

### 2.1. Subjects

This retrospective study recruited children under the age of 14 who were hospitalized in Hunan Provincial People's Hospital from January 2019 to December 2019 due to pneumonia. Moreover, they were diagnosed with AdV pneumonia and were treated with bronchoscopy alveolar lavage. The diagnostic criteria for AdV pneumonia included the following [11, 12]: (i) acute lower respiratory symptoms; (ii) chest X-ray or computed tomography (CT) showing lung infiltration; (iii) AdV infection determined by polymerase chain reaction (PCR) detection of respiratory viruses in bronchoalveolar lavage fluid (BALF). The exclusion criteria were as follows: (i) chronic lung disease; (ii) immunosuppressive or defective diseases; (iii) repeated respiratory infections; (iv) repeated wheezing or asthma; (v) bronchopulmonary dysplasia; (vi) severe organ diseases and malignant cancers; and (vii) incomplete case information. The study was approved by the Ethics Committee of Hunan Provincial People's Hospital with judgment under reference number 2020-07. All experiments were performed in compliance with relevant laws and institutional guidelines and in accordance with the ethical standards of the Declaration of Helsinki. Informed consent was obtained for any experimentation with human subjects.

### 2.2. AdV Sample Collection

Venous blood was drawn from all patients on the day of admission for functional testing, such as liver and kidney function, blood routine, and CRP. Respiratory pathogen testing samples mainly include sputum specimens, BALF, and nasopharyngeal swabs (NPAs). Sputum samples from infants and young children were collected by negative pressure suction, and sputum was expectorated from older children (>5 years old) in the morning for sputum culture.

BALF was obtained from children with AdV pneumonia included in this study. The specific method was as mentioned before [13]. In brief, BALF collection under fiberoptic bronchoscopy was completed within 1–7 days of the early course of the disease. BALF was placed in a 1.5 mL centrifuge tube and centrifuged at 12 000 r/min for 5 min. The pellet at the bottom of the tube was collected as a DNA extraction sample.

All nasopharyngeal swabs (NPAs) samples were collected within 24 h of admission. A disposable sterile suction tube was inserted 7–8 cm through the nasal cavity to achieve negative pressure below the pharynx to absorb 1–2 mL of deep NPAs. The collected samples were placed in sterile collection tubes. A small amount of normal saline and 2 mL of virus protection solution, containing 200 U/mL penicillin, streptomycin, amphotericin, and 0.125% bovine serum albumin, were added. After the above samples were mixed, they were immediately stored in a -80°C low-temperature refrigerator for the following testing.

### 2.3. Data Collection

Clinical data such as clinical manifestations, laboratory examinations, and diagnosis results were collected. Clinical information included gender, age, duration of hospitalization or fever, immunoglobulin, systemic corticosteroid, fiberoptic bronchoscopy lavage therapy, imaging reports of X-ray or CT, etc. Laboratory data contained blood routine, CRP, LD, D-dimer (DD), Alanine transaminase (ALT), creatine kinase MB (CK-M), humoral immunity, and cell-mediated immunity.

### 2.4. Real-Time PCR Detection of AdV

AdV-DNA replication multiples were detected using the real-time PCR method. The remaining BALF samples were kept at a low temperature. The kit was purchased from Qiagen (USA). The BALF sample was gently shaken for 30 s and centrifuged at 15000 × *g* for 5 min. Then, DNA was extracted according to the instructions of the kit, and the precipitate was collected from 400 *μ*l of the sample. The PCR instrument is the BIO-RADicycler gene amplification instrument (USA). AdV DNA>1.0 × 10^3^ copies/mL was positive.

### 2.5. Definition of Clinical Severity

The severity of pneumonia was classified according to the standards of the WHO guidelines. According to clinical characteristics, pneumonia could be divided into severe and nonsevere. Special treatment was given to each type of pneumonia. Patients with severe pneumonia received oxygen therapy or ventilator-assisted ventilation in Pediatric Intensive Care Unit (PICU). In children diagnosed with severe pneumonia, at least one of the following characteristics was present: central cyanosis, inability to breastfeed, drink, or vomit, convulsions, lethargy, unconsciousness, or severe respiratory distress [14].

### 2.6. Detection of Mixed Infections

NPAs were collected, and immunofluorescence was used to detect influenza A, influenza B, parainfluenza types 1, 2, and 3, and respiratory syncytial virus antigens (respiratory virus detection kit, Diagnostic Hybrids, USA). The processed sample was observed under a fluorescence microscope (×200). Positive staining showed that at least two intact cells had the fluorescent type of a certain virus. Other microorganisms, including typical bacteria, were detected by Gram staining and blood culture of sputum specimens. Atypical bacteria including *Chlamydia pneumoniae* and *Mycoplasma pneumoniae* were tested with antibodies in blood samples.

### 2.7. Statistical Analysis

SPSS25.0 statistical software was utilized for data analysis. The measured data conforming to the normal distribution were expressed as the mean ± standard deviation (*x* ± *s*). The independent sample *t*-test was used for comparison between the two groups. The median represented nonnormally distributed data, and the Wilcoxon rank sum test was used to compare the two groups. *P* < 0.05 was statistically significant. The enumeration data were expressed as a percentage, and the comparison between groups was performed by the *χ*^2^ test. Logistic regression analysis was performed on the risk factors related to BMP formation in the trachea of children with AdV pneumonia (variable selection criterion was *P* < 0.05, exclusion criterion was *P* > 0.1; detection level was bilateral; *α* = 0.05). In order to find the best predictor, the ROC curve was drawn. Risk factors were predictors. Sensitivity was on the ordinate, and 1-specificity was on the abscissa in the ROC curve. Pearson correlation analysis was performed.

## 3. Results

### 3.1. Patient Diagnosis and Characteristics

**Figure 1:**
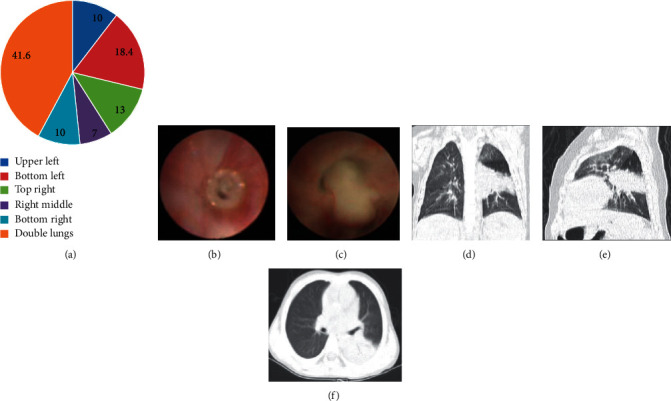
BMP formation distribution in the BMP group and the representative imaging features of a child with AdV pneumonia. (a) Distribution of mucus formation in the BMP group. (b, c) Representative pictures in bronchoscopy findings of the BMP of the annular stripping of the tracheal intima and the subbranch of the left lower basal branch. (d, e) The anteroposterior and lateral chest radiograph of the child. (f) The CT slice of the child.

Since Hunan Provincial People's Hospital is the largest children's bronchoscopy medical center in Hunan Province, a total of 453 children with AdV pneumonia received bronchoscopy treatment. They were divided into a BMP group and a control group. There were 185 cases (40.8%) in the BMP group and 268 cases (59.2%) in the control group. There were 112 (60.5%) and 179 (66.8%) men in the BMP group and control group, respectively, and the difference was not statistically significant (*P* > 0.05; Table 1). There was a substantial difference in the age between the BMP group and the control group (*P* = 0.004; Table 1). The study subjects were classified into six age groups, including less than 6 months, 6–23 months, 24–36 months, 37–48 months, 49–60 months, and more than 60 months (Figure 1(a)). The age of onset of children has a higher incidence within 6–23 months (*P* < 0.05). At the age of 6–23 months, the proportion of patients was markedly higher in the control group (54.68% vs. 35.14%, *P*<0.01; Table 1). Children clearly had longer length of hospitalization (*P* = 0.005; Table 1) and longer heat range throughout the disease period (10.5 ± 3.6 d vs. 8.2 ± 3.2 d, *P* = 0.001) in the BMP group. In addition, patients with BMP had a higher incidence of dyspnea, shortness of breath, ventilator-assisted ventilation, PICU admission, severe pneumonia, and progression to bronchiolitis obliterans (*P* < 0.05). In the laboratory test results, compared with the control group, the LDH, DD, and CRP levels of children in the BMP group were significantly increased (*P* = 0.003, *P* = 0.001, and *P* = 0.01, Table 1). The proportion of mixed *Mycoplasma pneumoniae* was considerably higher after BMP formation (54.6% vs. 32.5%, *P* < 0.001, Table 1). AdV load of the BALF was clearly elevated after BMP formation (6.47 ± 2.02 vs. 5.49 ± 1.98, *P* < 0.001, Table 1). Except for these parameters, there were no significant differences in allergic reactions, wheezing and liver enlargement symptoms, leukocyte (WBC), percentage of neutrophils (N%), hemoglobin (Hb), platelet (PLT), CK-MB, ALT, bacterial coinfection, total IgG, IgM, and CD3+, CD3+CD4+, and CD3+CD8+. Lung consolidation and pleural effusion cases were markedly greater after BMP formation. In addition, lung rales, mixed infection, and IgA in the BMP group were lower than those in the control group (*P* < 0.05) (Table 1). The lung inflammation mainly occurred in both lungs after BMP formation (77 out of 185 cases (41.6%)), followed by the left lower lung (34 cases (18.4%)) and the right upper lung (24 cases (13.0%)) (Figure 1(a)). There was no significant difference in the duration between the onset of symptoms and the start of treatment (corticosteroids, gamma globulin, and tracheoscopy intervention). There was no significant difference in the duration of fiberoptic bronchoscopy (Table 1). The performance of bronchoscopy and imaging of a 2-year-old AdV pneumonia child is shown in Figures 1(b)–1(f). Heat range, LDH, DD, *Mycoplasma* coinfection, and AdV load were the most significant differences between the two groups, suggesting potential risk factors for forming mucus plugs in childhood AdV pneumonia.

### 3.2. Infection of AdV Alone among Patients

To exclude the effects of mixed infection, we further conducted a study of the BMP formation in AdV pneumonia with AdV infection alone. There were 188 patients with AdV infection alone, including 64 (34.04%) in the BMP group and 124 (65.96%) in the control group. There was no significant difference in gender and age between the BMP and the control groups (*P* > 0.05, Table 2). The hospital stay and fever duration were longer in the BMP group (*P* < 0.05). In addition, the incidence of the allergic constitution, admission to PICU, ventilator-assisted ventilation, severe pneumonia, and progression to BO in patients with BMP was higher (*P* < 0.05). The laboratory test results showed that the DD levels were significantly higher in the BMP group than in the control group (*P*=0.002, Table 2). There was a significant difference in the AdV load between the two groups (7.14 ± 1.73 and 5.59 ± 1.84, *P* < 0.001, Table 2).

### 3.3. Predictive Risk Indexes

**Figure 2:**
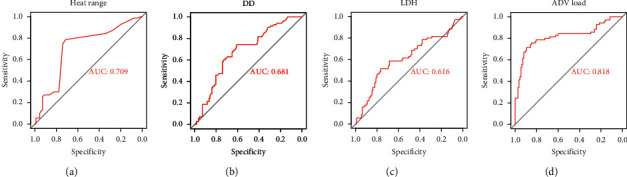
ROC curve analysis of patients with AdV infection. (a–c) The heat range, DD, and LDH in the ROC curve, which predicted the sensitivity and specificity of AdV pneumonia-BMP formation at 78.6% and 71.4%, 74.3% and 60.7%, and 51.4% and 76.8%, respectively. (d) The AdV load in the ROC curve, which predicted the sensitivity and specificity of AdV pneumonia--BMP formation at 71.4% and 89.3%, respectively.

To further determine the risk factors for BMP formation in children with AdV pneumonia, Univariate logistic regression was used to analyze. The result showed that in the BMP group, AdV load (odds ratio (OR), 3.380; 95% confidence interval (CI), 2.082–5.488), *Mycoplasma* coinfection (OR, 1.536; 95% CI, 1.363–1.731), serum DD level (OR 1.990, 95% CI 1.204–3.287), heat range (OR, 3.308; 95% CI, 1.302–8.403), and serum LDH level (OR, 1.603; 95% CI, 1.000–2.568) were independent risk factors for AdV pneumonia-BMP formation, respectively (*P* < 0.05, Table 3). ROC curve analysis showed that heat range, DD, LDH, and AdV load independently predicted the AUC of AdV pneumonia-BMP to be 0.709, 0.681, 0.616, and 0.818, severally. The sensitivity and specificity of heat range, DD, and LDH in predicting AdV pneumonia-BMP formation were 78.6% and 71.4%, 74.3% and 60.7%, 51.4% and 76.8%, respectively (Table 3 and Figures 2(a)–2(c)). The best cut-off value of AdV load was 6.76, and the sensitivity and specificity of predicting AdV pneumonia-BMP formation were 71.4% and 89.3%, respectively (Table 3 and Figure 2(d)).

**Figure 3:**
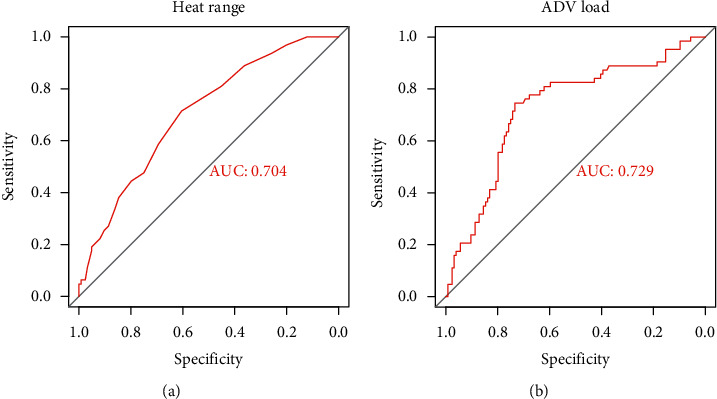
ROC curve analysis of patients with a single AdV infection. (a, b) The heat range and AdV load in the ROC curve predicted the sensitivity and specificity of single AdV pneumonia BMP formation at 71.4% and 60.5% and 74.6% and 73.4%, respectively.

To analyze the effects of AdV infection alone on pneumonia, we performed a risk factor analysis of single AdV-infected patients with BMP formation. The results showed that AdV load (OR, 1.522; 95% CI, 1.038–1.175) and heat range (OR, 1.104; 95% CI, 1.249–1.853) were independent risk factors for single AdV-infected patients of the BMP formation (*P* < 0.05, Table 4). ROC curve analysis showed that the AUC of AdV load and heat history independently predicted the BMP formation at 0.704 and 0.729 (Figures 3(a)–3(b)). The best cut-off value for AdV load was 6.76, and the sensitivity and specificity for predicting AdV pneumonia-BMP formation were 74.6% and 73.4%, respectively (Table 4 and Figure 3(b)).

**Figure 4:**
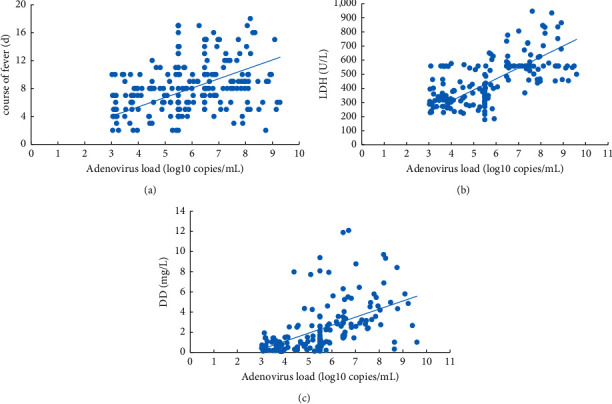
Correlation analysis among AdV load, heat range, LDH, and DD in the BMP group of children with AdV infection. (a) Correlation curve between AdV load and heat range in the BMP group (*R* = 0.309, *P*=0.003). (b) Correlation curve between AdV load and LDH in the BMP group (*R* = 0.644, *P*=0.001). (c) Correlation curve between AdV load and DD in the BMP group (*R* = 0.531, *P*=0.002).

AdV load was positively correlated with DD, LDH, and heat range, respectively (*R* = 0.531, *P*=0.002; *R* = 0.644, *P* = 0.001; *R* = 0.309, *P*=0.003) (Figure 4). The above results suggested that AdV load might be the risk factor for BMP formation in children with AdV pneumonia.

## 4. Discussion

In the study, we described the characteristics of AdV pneumonia in children and explored the risk factors for BMP formation. In the research, the proportion of BMP with AdV infection was higher, which might be related to the outbreak of AdV in southern China in 2019 and the timely use of fiberoptic bronchoscopy. The humid and cold environment in southern China is conducive to the survival of AdV pneumonia. BMP can be as high as 30% in refractory *Mycoplasma pneumoniae* pneumonia [15], but related studies on BMP formation after AdV infection are lacking. The damage to the bronchial mucosa of different pathogens is different, so the occurrence of BMP will also be different in the prevalence of AdV and *Mycoplasma pneumoniae* pneumonia.

The study showed that AdV load had a strong predictive effect on the formation of BMP with AdV pneumonia. The best cut-off value of AdV load for prediction of BMP was 6.76 (log10 copies/mL). The sensitivity and the specificities were 71.4% and 89.3%, respectively. We conducted a risk factor analysis of AdV pneumonia-BMP with AdV-infected alone. AdV load independently predicted the AUC of AdV pneumonia-BMP was 0.729, and the optimal cut-off value of the load was 6.76. The sensitivity and specificity of predicting AdV pneumonia-BMP were 74.6% and 73.4%, respectively. Previous studies have shown that AdV load is closely related to the severity and prognosis of AdV pneumonia. For example, Goikhman Y et al. retrospectively analyzed 123 children with AdV pneumonia and found that AdV load is closely related to the severity of AdV pneumonia [16]. Leyun Xie et al. found that AdV load can reflect the seriousness of AdV pneumonia, indicating that AdV load level may be a risk indicator to predict the progression of severe AdV pneumonia [11]. In this study, when the AdV load in AdV pneumonia was greater than 6.76, the risk of BMP formation increased.

Previous studies have shown that age is a risk factor for BMP in *Mycoplasma* pneumonia [15]. Children with mixed *Mycoplasma*-forming BMP were relatively older [9]. Our study found that between the BMP group and the control group of AdV pneumonia, children aged 6–23 months were the most affected. The median age of the BMP group was higher than that of the control group, which might be related to mixed infections, especially the higher proportion of mixed *Mycoplasma* infections. The age might be related to the formation of BMP in AdV pneumonia. Our study showed that the BMP group had a significantly longer heat range and hospital stay than the control group. The proportion of dyspnea, ventilator-assisted ventilation, lung consolidation, complications, and progression to severe pneumonia and bronchiolitis obliterans in the BMP group was significantly increased. Among them, the heat range is a risk factor for AdV pneumonia, consistent with the study of Xu et al. [17]. However, ROC curve analysis showed that the AUC of the AdV pneumonia-BMP independently predicted by the heat range was significantly lower than the predictive effect of AdV load. Long-term fever indicates that inflammation persists and has not been effectively controlled. AdV load was positively associated with heat range, indicating that the higher the AdV load, the longer the duration of inflammation. Long-term inflammatory stimulation can cause tracheal mucosal damage and loss of necrosis, increase mucus secretion, and accelerate BMP formation [18].

In the study, we found that LDH and DD levels were related to the formation of AdV pneumonia-BMP. ROC curve analysis of patients with AdV infection showed that the AUC of LDH and DD was 0.619 and 0.681, respectively. Although they were not independent risk factors for the BMP formation in AdV infection alone, they still had important implications for clinical research. Serum LDH not only reflects the degree of inflammation in the body but also the degree of damage to lung tissue, so LDH level is also one of the indicators for evaluating the severity of pneumonia [19]. LDH is markedly increased in severe AdV pneumonia [20]. The LDH of the BMP group was significantly increased, and it was also a risk factor for BMP occurrence in our study, as well as the study of Zhang et al. [21]. Correlation analysis showed a significant positive correlation between AdV load and LDH, indicating that higher AdV load levels might be conducive to the formation of more inflammatory factors and BMP. Different from previous studies, the study showed that DD levels were related to the formation of BMP in AdV pneumonia. DD is a fibrin-specific degradation product produced by plasmin hydrolysis. An increase in the level of D-dimer indicates that the body is in a hypercoagulable state, which promotes the formation of BMP [22]. Relevant studies have found that DD is significantly increased in CAP patients requiring hospitalization, and it is related to disease severity and survival rate [23]. In addition, the plasma D-dimer level of patients with complications was significantly higher than that of patients without complications [24]. Therefore, the plasma DD level can predict the severity of pneumonia and prognosis [25]. In the study, Pearson correlation analysis also found a significant positive correlation between AdV load and DD, indicating that high-level replication of AdV might be beneficial to promoting the formation of hypercoagulable state and BMP.

Previous studies have shown that compared with simple AdV pneumonia, children with a mixed virus or bacterial pathogens with AdV pneumonia have no difference in clinical characteristics and disease severity [11]. However, the results of this study indicate that mixed infections, especially mixed *Mycoplasma* infections, were a risk factor for BMP formation in children with AdV pneumonia. It is speculated that mixed infections might have a synergistic effect on the formation of BMP and accelerate the pathophysiological response. Although BMP formation is an important cause of *Mycoplasma pneumoniae* pneumonia [15], the relationship between mixed *Mycoplasma* infection with AdV pneumonia in children and BMP formation has not been reported.

The pulmonary innate and adaptive immune responses are also one of the main predictive parameters during pneumonia [26]. Hyperfunction of IFN-*γ*-secreting T cells, especially CD8+T cells, may be involved in the pathogenesis and severity of AdV pneumonia [27]. In this study, humoral and cellular immunity data were analyzed. The results showed no significant differences in total IgG, IgA, IgM, CD3+, CD3+CD4+, and CD3+CD8+ between the control group and the BMP group, whether AdV infection alone or mixed infection AdV pneumonia. In patients with AdV-infected pneumonia alone, immune-related indicators (total IgA, CRP, and LDH) were not significantly different between the two groups. Therefore, we speculated that humoral and cellular adaptive immune responses might not be key risk factors for BMP formation in patients with AdV pneumonia. The pathogenesis of severe disease induced by human AdV-7 may be related to high replication capacity and high inflammatory response [28]. AdV may be closely associated with a strong inflammatory response in pediatric patients [29]. Zhang et al. showed that CRP and LDH levels were independent risk factors for BMP formation [9]. In the risk analysis of BMP formation in coinfection with AdV pneumonia, there were also differences in CRP and LDH levels. However, in patients with AdV-infected pneumonia alone, immune-related indicators (total IgA, CRP, and LDH) were also not significantly different between the two groups. We speculated that the difference between the previous study and our study might be related to the infection of AdV mixed *Mycoplasma* pneumoniae and other pathogens in AdV pneumonia. Xu et al. believed that age and IL-10 and IFN-*γ* levels have important predictive value for BMP formation [10]. However, since the detection of other innate immune inflammatory indicators is not a routine clinical detection item, it is difficult to obtain. The corresponding analysis was not performed. This is a limitation of our study. In future studies, we plan to explore further the impacts of immune responses on BMP formation in AdV pneumonia.

The study showed that the starting time of glucocorticoids was related to BMP formation [21]. However, there were no marked differences in the onset of glucocorticoid use, the time of fiberoptic bronchoscopy intervention, and the time of use of gamma globulin between the BMP group and the control group in children with AdV pneumonia. The study was a retrospective study, so there might be some selection bias. In addition, the study has the limitation of a relatively single sample size. The study sample was only from Hunan Province, China. The scope of the study is limited. The sample size of the study is not very large. There might be regional differences in research subjects. Therefore, recruiting more patients with AdV pneumonia from different regions is necessary for a further prospective study.

In conclusion, the study illustrated that AdV load could be an independent predictor of mucus thrombus formation in children with AdV pneumonia. Especially when AdV load is>5.785, the risk of mucus thrombus formation in children with AdV pneumonia increases significantly with the increased AdV load.

## Figures and Tables

**Table 1:** Comparison of the clinical characteristics of patients with AdV pneumonia in the BMP and control group.

Variables	BMP group (*n* = 185)	Control group (*n* = 268)	*P* value
Characteristics			
Gender (male)	112 (60.54)	179 (66.79)	0.172
Age (M(P25–P75))/(months)	36 (12–60)	24 (12–42)	0.004

Age group (months)			
<6	5 (2.70)	10 (3.73)	0.548
6–23	65 (35.14)	146 (54.48)	<0.001
24–36	37 (20.00)	35 (13.06)	0.047
37–48	24 (12.97)	32 (11.94)	0.743
49–60	21 (11.35)	19 (7.09)	0.116
>60	35 (18.92)	24 (8.96)	0.002
Length of hospitalization (M(P25–P75))/d	10 (8–16)	9 (7–14)	0.005
Allergic constitution (*n* (%))	18 (9.73)	21 (7.84)	0.480

Signs and symptoms			
Dyspnea (*n* (%))	49 (26.49)	50 (17.48)	0.047
Heat range (x ± s)/(d)	10.52 ± 3.60	8.18 ± 3.16	0.001
Tachycardia (*n* (%))	119 (64.32)	146 (51.05)	0.037
Lung rales (*n* (%))	152 (82.16)	232 (86.56)	<0.001
Lung wheezing (*n* (%))	25 (13.51)	45 (16.79)	0.343
Hepatomegaly (*n* (%))	53 (28.65)	66 (23.08)	0.339
Poor spirit (*n* (%))	22 (11.89)	15 (5.60)	0.016
Complication (*n* (%))	70 (37.84)	75 (26.22)	0.027

Disease severity			
Mild (*n* (%))	101 (54.59)	188 (70.15)	0.001
Severe (*n* (%))	84 (45.40)	80 (29.85)	0.001
Admission to PICU (*n* (%))	44 (23.78)	31 (11.57)	0.001
Ventilator-assisted ventilation (*n* (%))	50 (27.03)	50 (18.66)	0.035
Bronchiolitis obliterans (*n* (%))	23 (12.43)	9 (3.36)	<0.001

Laboratory characteristics			
WBC (M(P25–P75))/×109 L−1	7.03 (4.56–9.38)	7.34 (5.45–10.55)	0.156
N% (M(P25–P75))/%	49.70 (41.35–66.6)	48.9 (34.1–61.3)	0.103
Hb (M(P25–P75))/g·L−1	109 (98.5–118)	110 (101–119)	0.173
PLT (M(P25–P75))/×109 L−1	276 (172.5–383.5)	281 (202–399)	0.568
CRP (M(P25–P75))/mg·L−1	9.28 (3.13–26.37)	7.38 (3.13–20.37)	0.01
LDH (M(P25–P75))/U·L−1	463 (349–634)	406 (325–552)	0.003
DD (M(P25–P75))/mg·L−1	1.43 (0.62–3.20)	0.73 (0.41–1.7)	0.001
CK-MB (M(P25–P75))/ng·mL−1	29 (23–40.25)	28 (21–41)	0.440
ALT (M(P25–P75))/U·L−1	21.4 (14.8–33.46)	18.1 (12–34.75)	0.583
Mixed infection (*n* (%))	121 (65.41)	144 (53.73)	0.015
Bacterial coinfection (*n* (%))	29 (15.68)	47 (16.43)	0.602
*Mycoplasma* coinfection (*n* (%))	101 (54.59)	93 (32.52)	<0.001
AdV load from BALF (x ± s), log10 copies/mL	6.47 ± 2.02	5.49 ± 1.98	<0.001

Humoral immunity (M(P25–P75), %)			
IgG (M(P25–P75))/g·L−1	8.72 (6.96–10.90)	8.02 (6.36–9.72)	0.214
IgA (M(P25–P75))/g·L−1	0.73 (0.44–1.22)	0.96 (0.7–1.36)	0.026
IgM (M(P25–P75))/g·L−1	1.06 (0.69–1.42)	0.98 (0.71–1.38)	0.535

Cellular immunity (M(P25–P75), %)			
CD3+	55.63 (45.3–63.13)	56.67 (46.55–65.89)	0.562
CD3+CD4+	26.86 (18.98–32.83)	27 (21.93–36.18)	0.936
CD3+CD8+	21.98 (17.55–29.35)	20.28 (17.36–28.02)	0.737

Radiological characteristics			
Lung consolidation (*n* (%))	95 (51.35)	105 (36.71)	0.01
Pleural effusion (*n* (%))	11 (5.95)	5 (1.75)	0.021

Treat time (M(P25–P75))/day of course			
Corticosteroids	9 (7–11)	8 (6–10)	0.673
Gamma globulin	8 (5–9)	7 (4–9)	0.904
Tracheoscopy intervention	10 (6–14)	9 (5–14)	0.131

BMP, bronchial mucus plug; AdV, Adenovirus; WBC, leukocyte; N%, percentage of neutrophils; Hb, hemoglobin; PLT, platelet; CRP, C-reactive protein; LDH, lactate dehydrogenase; DD, D-dimer; CK-MB, creatine kinase isoenzyme; ALT, gluten laboratory data of alanine transaminase; IgA, immunoglobulin A; IgG, immunoglobulin G; IgM, immunoglobulin M.

**Table 2:** Comparison of the clinical characteristics of patients with single AdV infection in the BMP and control group.

Variables	BMP group (*n* = 64)	Control group (*n* = 124)	*P* Value
Characteristics			
Gender (male)	36 (56.25)	87 (70.16)	0.057
Age (M(P25–P75))/(months)	26 (12–48)	20 (10–36)	0.31

Age group (months)			
<23	27 (42.19)	69 (55.65)	0.08
24–60	30 (46.88)	46 (37.10)	0.19
>60	6 (9.38)	9 (7.26)	0.61
Length of hospitalization (M(P25–P75))/d	10 (7–15)	8 (6–12)	0.044
Allergic constitution (n (%))	13 (20.31)	6 (4.84)	0.001

Signs and symptoms			
Dyspnea (n (%))	30 (46.88)	45 (36.29)	0.16
Heat range (M(P25–P75))/d	12 (9–17)	8 (6–12)	<0.001
Tachycardia (n (%))	42 (65.63)	66 (53.23)	0.103
Lung rales (n (%))	58 (90.63)	107 (86.29)	0.39
Lung wheezing (n (%))	21 (32.81)	30 (24.19)	0.208
Hepatomegaly (n (%))	28 (43.75)	37 (29.84)	0.057
Poor spirit (n (%))	26 (40.63)	30 (24.19)	0.02
Complication (n (%))	32 (50)	37 (29.84)	0.007

Disease severity			
Mild (n (%))	39 (60.94)	102 (82.26)	0.001
Severe (n (%))	25 (39.06)	22 (17.74)	0.001
Admission to PICU (n (%))	22 (34.38)	20 (16.13)	0.004
Ventilator-assisted ventilation (n (%))	9 (14.06)	6 (4.84)	0.027
Bronchiolitis obliterans (n (%))	9 (14.06)	5 (4.03)	0.013

Laboratory characteristics			
WBC (M(P25–P75))/×10^9^ L^−1^	8.83 (6.36–13.35)	10.28 (7.49–14.24)	0.48
N% (M(P25–P75))/%	39.8 (34.65–58.7)	41.3 (27.5–59.5)	0.98
Hb (M(P25–P75))/g·L^−1^	112 (108–115)	115 (108–122)	0.57
PLT (M(P25–P75))/×10^9^ L^−1^	280 (199–411)	350 (253–499)	0.26
CRP (M(P25–P75))/mg·L^−1^	12 (3–26)	6 (3–15)	0.36
LDH (M(P25–P75))/U·L^−1^	407 (327–612)	367 (272–430)	0.13
DD (M(P25–P75))/mg·L^−1^	1.58 (0.77–2.68)	0.7 (0.4–1.54)	0.002
CK-MB (M(P25–P75))/ng·mL^−1^	29 (22–42)	30 (21–42)	0.82
ALT (M(P25–P75))/U·L^−1^	24 (14–42)	20 (14–33)	0.57
AdV load from BALF (*x* ± *s*), log10 copies/mL	7.14 ± 1.73	5.59 ± 1.84	<0.001

Humoral immunity (M(P25–P75), %)			
IgG (M(P25–P75))/g·L^−1^	7.85 (6.41–9.95)	8.04 (6.4–9.84)	0.39
IgA (M(P25–P75))/g·L^−1^	0.93 (0.58–1.2)	0.77 (0.44–1.21)	0.68
IgM (M(P25–P75))/g·L^−1^	1.04 (0.67–1.45)	0.95 (0.76–1.33)	0.18

Cellular immunity (M(P25–P75), %)			
CD3+	58.14 (47.4–64.8)	52.63 (45.54–71.13)	0.76
CD3+CD4+	27 (20.56–36.08)	27 (22.28–35.58)	0.5
CD3+CD8+	20 (18.96–27.38)	22.52 (18.71–29.03)	0.81

Radiological characteristics			
Lung consolidation (n (%))	40 (62.5)	86 (69.35)	0.343
Pleural effusion (n (%))	4 (6.25)	6 (4.84)	0.683

Treat time (M(P25–P75))/day of course			
Corticosteroids	7 (6–11)	7 (6–10)	0.69
Gamma globulin	7 (5–8)	7 (4–11)	0.62
Tracheoscopy intervention	9 (6–12)	9 (5–12)	0.73

BMP, bronchial mucus plug; AdV, Adenovirus; WBC, leukocyte; N%, percentage of neutrophils; Hb, hemoglobin; PLT, platelet; CRP, C-reactive protein; LDH, lactate dehydrogenase; DD, D-dimer; CK-MB, creatine kinase isoenzyme; ALT, gluten laboratory data of alanine transaminase; IgA, immunoglobulin A; IgG, immunoglobulin G; IgM, immunoglobulin M.

**Table 3:** Logistic regression analysis of patients with AdV infection.

Variable	Partial regression coefficient (*β*)	SE	Wald *χ*^2^	*P*	OR	95% CI
AdV load (log10 copies/mL)	1.218	0.247	24.266	<0.001	3.380	2.082–5.488
*Mycoplasma* coinfection	0.429	0.061	49.497	<0.001	1.536	1.363–1.731
DD (mg/L)	0.688	0.256	7.210	0.007	1.990	1.204∼3.287
Heat range/d	1.196	0.476	6.328	0.012	3.308	1.302–8.403
LDH (U/L)	0.472	0.240	3.861	0.049	1.603	1.001–2.568
Constant	−3.404	0.408	69.754	<0.001	0.033	

AdV, adenovirus; DD, D-dimer; LDH, lactate dehydrogenase; SE, standard error; OR, odds ratio; CI, confidence interval.

**Table 4:** Logistic regression analysis of patients with a single AdV infection.

Variable	Partial regression coefficient (*β*)	SE	Wald *χ*^2^	*P*	OR	95% CI
AdV load (log10 copies/mL)	0.420	0.100	17.443	0.000	1.522	1.038–1.175
Heat range/d	0.099	0.032	9.840	0.002	1.104	1.249–1.853
Constant	−4.391	0.758	33.594	0.000	0.012	

AdV, adenovirus; SE, standard error; OR, odds ratio; CI, confidence interval.

## Data Availability

All data generated and analyzed during this study are included in this article.

## Supplementary Materials

Supplementary Materials
*Supplementary Table 1*. Pneumonia pathogens and infection ratio statistics.
