# Supplementary material for: Predictive Value of Adenoviral Load for Bronchial Mucus Plugs Formation in Children with Adenovirus Pneumonia
Source: Can Respir J. 2022 Aug 8;2022:9595184. doi: 10.1155/2022/9595184 (PMC9377942; doi:10.1155/2022/9595184)
Supplement: Supplementary Materials — Supplementary Table 1. Pneumonia pathogens and infection ratio statistics. [file 9595184.f1.docx]

| **Supplementary Table 1.** Pneumonia pathogens and infection ratio statistics. | | |
| --- | --- | --- |
| **Respiratory pathogen** | **Percentage** | **Most recent citations (PMID)** |
| **Virus** |  |  |
| Adenovirus | 7%-19% | 35361166; 30882722; 28975629 |
| Respiratory syncytial virus | 7.9%-31.2% | 35361166; 30882722; 28975629 |
| Parainfluenza virus | 5%-23.2% | 35361166; 30882722; 28975629 |
| Rhinovirus | 21.10% | 35361166 |
| Influenza virus | 2.3%-18.3% | 35361166; 30882722; 28975629 |
| **Bacteria** |  |  |
| Staphylococcus aureus | 2.1%-3% | 35361166; 35072019 |
| Streptococcus pneumoniae | 11.2%-36% | 32629486; 35361166 |
| **Mycoplasma pneumoniae** | 13.9%-32.4% | 35361166;28975629 |
| Others | 16% | 35361166; |
